# Dietary magnesium is able to influence the relationship between vitamin C and estimated glomerular filtration rate: A cross‐sectional study

**DOI:** 10.1002/fsn3.3456

**Published:** 2023-06-01

**Authors:** Zheng‐yang Lin, Yong‐yi Liang, Ru Wang, Biao Hu, Wen‐ju He, Jun‐kui Li, Zi‐ang Ding, Zhuo‐yuan Lin, Shi Zhang

**Affiliations:** ^1^ Department of Clinical Medicine The Second Clinical School of Guangzhou Medical University Guangzhou China; ^2^ Department of Preventive Medicine School of Public Health, Guangzhou Medical University Guangzhou China; ^3^ Department of Medical Imaging The Second Clinical School of Guangzhou Medical University Guangzhou China; ^4^ Department of Clinical Medicine The First Clinical School of Guangzhou Medical University Guangzhou China; ^5^ Department of Urology The Second Affiliated Hospital of Guangzhou Medical University Guangzhou Medical University Guangzhou China; ^6^ Department of Gastrointestinal Surgery The Second Affiliated Hospital of Guangzhou Medical University Guangzhou China

**Keywords:** dietary magnesium, dietary vitamin C, estimated glomerular filtration rate (eGFR), interaction

## Abstract

The estimated glomerular filtration rate (eGFR) is a comprehensive index that is widely used to assess renal function. Although studies have confirmed a correlation between eGFR and dietary vitamin C, the impact of varying levels of vitamin C on eGFR remains unclear. Additionally, the interaction between dietary magnesium intake and vitamin C concentration on eGFR is not well understood. As such, the objective of this study was to investigate the relationship between dietary magnesium intake and vitamin C in relation to eGFR. This study analyzed the data of consecutive NHANES from 2005 to 2018. We included 17,633 participants aged 20 or older and used multiple linear regression analysis to evaluate the relationship between dietary vitamin C and eGFR. Dietary Mg intake from experimental data was dichotomized into a low dietary Mg intake group (≤254 mg/day) and a normal dietary Mg intake group (>254 mg/day). To evaluate the impact of dietary magnesium intake on eGFR, a multivariable linear regression was conducted utilizing an interaction test between dietary vitamin C and eGFR. We discovered a positive association between dietary vitamin C content and eGFR. The relationship between dietary vitamin C levels and eGFR differed between individuals with low Mg intake and those with normal Mg intake (*β*: 2.96 95% CI:1.63 ~ 4.29 vs. *β*: 1.05 95% CI: −0.15 to 2.25), and the positive association of high dietary vitamin C content with eGFR was stronger in the low Mg intake group. Furthermore, we observed that dietary magnesium intake significantly altered the positive association between dietary vitamin C and eGFR (interaction value of 0.020). Our experimental study revealed that the interaction between dietary magnesium and dietary vitamin C can significantly impact eGFR. This finding carries significant implications for the treatment of diseases resulting from abnormal eGFR, as well as the selection of clinically relevant drugs.

## Graphical abstract

Glomerular filtration rate is the most useful measure of renal function, which is affected by several factors. Our study found that vitamin C intake would affect GFR, and dietary Mg intake would further affect the relationship between vitamin C and GFR.

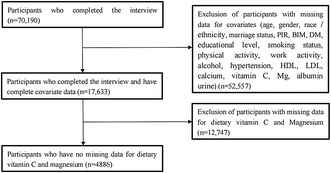

## 1 INTRODUCTION

Glomerular filtration rate (eGFR) is considered the most useful overall indicator of renal function (Helal et al., 2012; Stockand & Sansom, 1997). As individuals age, renal aging becomes more common and is characterized by intimal fibrosis and glomerulosclerosis, renal tubular atrophy, and interstitial fibrosis. This process can lead to a gradual decline in eGFR (Abdelhafiz et al., 2010). However, numerous primary diseases, such as hypertension, diabetes, and cardiovascular diseases, can cause harm to the structure and function of the kidneys under pathological conditions. For instance, diabetes can damage the microvasculature, leading to diabetic nephropathy (DN), which is a major contributor to end‐stage renal disease (Pradeep et al., 2019). At the same time, diabetes is considered to modify the prognosis of kidney disease as much as hypertension (James et al., 2015). Cardiovascular disease (CVD) is accompanied by related renal artery, artery, and capillary (glomerular) injury, resulting in renal ischemia and a progressive decline in the renal function that manifest with the reduction in the eGFR (Ishani et al., 2009). In summary, eGFR evaluation holds great significance for the diagnosis and prognosis of kidney diseases (Ferguson et al., 2016). A thorough comprehension of eGFR can enable clinicians to administer appropriate medications, fluids, and early interventions to prevent end‐stage renal failure (Work & Schwartz, 2008).

Ascorbic acid, also known as vitamin C, is an essential nutrient and cofactor in several enzyme pathways. Its main dietary source is fresh fruits and vegetables (Ferraro et al., 2016). Renal insufficiency is associated with a decrease in plasma vitamin C, which is an essential nutrient and cofactor in several enzymatic pathways (Ferguson et al., 2016). Accordantly, in a meta‐analysis involving four randomized controlled trials (RCTs) and 195 participants, antioxidant therapy significantly improved kidney function (defined as estimated glomerular filtration rate, eGFR) (Jun et al., 2012). Moreover, a prospective observational cohort study involving patients with severe sepsis or septic shock revealed that the concomitant administration of ascorbate significantly reduced the risk of acute kidney injury (AKI) caused by colistin administration (Dalfino et al., 2015). However, a pooled analysis of three randomized controlled trials involving 85 patients with diabetic kidney disease showed that antioxidant supplements had no significant impact on GFR compared to the control group. This suggests that other unknown confounding factors may have a significant influence on the kidneys' protective efficacy of antioxidant therapy (Bolignano et al., 2017). Therefore, the effect of dietary vitamin C on renal function needs to be further verified.

Magnesium is an essential cofactor for various enzymes and is involved in several metabolic pathways, such as glycolysis, DNA synthesis and transcription, protein synthesis, intracellular signal transduction, regulation of ion channels producing intracellular ion currents, and determination of membrane voltage (Tangvoraphonkchai & Davenport, 2018). As a result, magnesium plays a crucial role in multiple fundamental physiological functions of vascular smooth muscle cells and endothelial cells (Sakaguchi et al., 2018). Previous data have indicated that magnesium deficiency is linked to a higher risk of cardiovascular diseases and chronic kidney disease (Sakaguchi et al., 2018). Furthermore, magnesium has been shown to counteract phosphate‐induced apoptosis of vascular smooth muscle cells and prevent vascular calcification. Additionally, it can protect against renal injury caused by hyperphosphatemia (Sakaguchi et al., 2015). A retrospective cohort study of 311 patients with non‐dialysis chronic kidney disease (CKD) evinced that patients with high phosphate and low magnesium levels are at an increased risk of end‐stage kidney disease (ESKD) compared to the high phosphate and high magnesium groups (Sakaguchi et al., 2015). Moreover, hypomagnesemia has been implicated in the development of complications, such as diabetes mellitus and graft loss, after kidney transplants (Sakaguchi et al., 2015). These data support the renal protective roles of magnesium. Previous experiments have demonstrated that both magnesium and vitamins possess protective effects on renal function. However, it remains unclear whether there is an interaction between magnesium and vitamin C. Our aim was to investigate whether dietary magnesium and vitamin C interact to influence renal function. To achieve this, we conducted a cross‐sectional study to explore the relationship between dietary vitamin C concentrations and eGFR, and the effect of dietary magnesium intake on this relationship. As dietary magnesium and vitamin C are common components in daily diets, easily accessible, and have no side effects within normal ranges, both of them can be supplemented or adjusted in the early stages of reduced kidney function to prevent and control diseases. They can also play a supportive role in the treatment of kidney diseases, providing new ideas for the prevention and treatment of kidney diseases in clinical practice.

## 2 METHODS

### 2.1 Data source

The study used seven stages of the National Health and Nutrition Examination Survey (NHANES): 2005–2006, 2007–2008, 2009–2010, 2011–2012, 2013–2014, 2015–2016, and 2017–2018. NHANES conducted a cross‐sectional survey of the noninstitutionalized civilian population in the United States, providing a nationally representative sample. Health interviews were conducted at participants' homes to gather demographic, socioeconomic, and health‐related information, while extensive physical examinations were performed at the Mobile Examination Center (MEC). Initially, data on 70,190 recruited participants were obtained for this study. After eliminating missing data for potential covariates, our analysis included 17,633 participants. Notably, 12,747 participants with missing data on serum magnesium and vitamin C levels were excluded. Finally, our analysis included 4886 participants with complete data for further investigation. Furthermore, researchers did not observe any additional discomfort among these participants, and all participants provided written informed consent before participating in the investigation. This study was approved by the research ethics review board of the NCHS.

### 2.2 Measurement of vitamin C content

Data on dietary vitamin C intake were obtained from the NHANES database, which uses 24‐hour dietary intake survey methods, a consensus reached by the NHANES panel at regular workshops, to evaluate methods for data collection in NHANES. This recall method has continued in NHANES over the years (Ahluwalia et al., 2016). As part of our study, we collected data on participants' dietary vitamin C intake over the past 24 h through the MEC dietary review interview. This intake was then categorized as either high or low based on dietary vitamin C content.

### 2.3 Determination of dietary magnesium intake

A precise inventory of all meals consumed by the participant in the previous 24 h was used to obtain data on dietary magnesium intake. For this study, the first 24 h of dietary information for participants was derived from a 24‐h dietary recall interview personally collected by the MEC. Based on dichotomization, daily dietary magnesium intake was divided into the normal dietary Mg intake group (>254 mg/day) and the low dietary Mg intake group (≤254 mg/day).

### 2.4 Measurement of glomerular filtration rate

Participants' eGFR data were obtained from NHANES, and the estimation of GFR in NHANES was performed with a creatinine clearance based method (Sakaguchi et al., 2018), using the formula CKD‐EPI‐Scr.

### 2.5 Covariates

Our analytical model incorporated potential covariates such as age, gender, race/ethnicity, marital status, poverty income ratio (PIR), body mass index (BMI), education level, smoking status, physical activity, work activity, alcohol consumption, diabetes mellitus (DM), hypertension, urine albumin, low‐density lipoproteins (LDL), and high‐density lipoproteins (HDL). Race was categorized as Mexican Americans, non‐Hispanic blacks, non‐Hispanic whites, other Hispanics, and other races (including multiple races). Marital status was divided into unmarried, married (including separation, divorce, cohabitation, marriage, widowhood), and unknown. To evaluate household income, we utilized the poverty income ratio (PIR), which is determined by family size specification, and calculated the threshold accordingly. BMI was categorized into three intervals: <25, 25–29.9, and >30. Education level was divided into below high school, high school graduation, university, and above. Smoking status was classified as current smoker (lifetime smoking of more than 100 cigarettes, smoking for a few days, or every day), former smoker (smoking more than 100 cigarettes in a lifetime but currently not smoking), never smoked (smoking less than 100 cigarettes in a lifetime), and unknown. Physical activity was defined based on the level of intensity, ranging from no or unknown activity to medium‐intensity sports, fitness, or entertainment activities, and high‐intensity sports, fitness, or entertainment activities. The work activities were categorized into four groups: no work activities, moderate work activities, intense work activities, and unknown. Alcohol consumption data were collected through questionnaire interviews and were grouped into at least 12 alcoholic beverages per year, less than 12 alcoholic beverages per year, and unknown. The diagnostic criteria for diabetes included: doctors confirming the patient's diabetes status, patient‐reported diabetes, glycoprotein HbA1c (%) >6.5, fasting blood glucose (mmol/L) = 7, random glucose (mmol/L) >11.1, two‐hour oral glucose tolerance test blood glucose (mmol/L) = 11.1, and the use of diabetes medication or insulin. The diagnostic criteria for hypertension included: the use of hypertension medication, the actual measured blood pressure value, the doctor's diagnosis, and self‐reported hypertension in the questionnaire. Additionally, the NHANES laboratory test data provided specific information on urinary albumin, LDL, and HDL concentration.

### 2.6 Statistical analysis

The statistical package R (http://www.R‐project.org, R Foundation) and free statistical software version 1.3 were used for all data analyses. During statistical analysis, we extracted stratified sampling data from the NHANES database to detect the relationship between dietary vitamin C and eGFR by employing a multiple linear regression procedure. A linear trend test was performed by entering the median value of each dietary vitamin C as a continuous variable in the model. The mean values of eGFR were assessed across the strata of magnesium intake. Interactions between subgroups were examined by the likelihood ratio test. The level of statistical significance was set at *p* < .05. Furthermore, 95% confidence intervals (CI) were also calculated. The continuous variables were presented with mean and standard deviation (SD) or median and interquartile range (IQR), and the categorical variables were presented with weighted percentages (%) in the descriptive analysis. The chi‐square test (categorical variables) and *t*‐test (normal distribution), and the Kruskal–Wallis (skewed distribution) test were used to evaluate the continuous and categorical variables, respectively.

## 3 RESULTS

### 3.1 Baseline characteristics of the study participants

**FIGURE 1:**
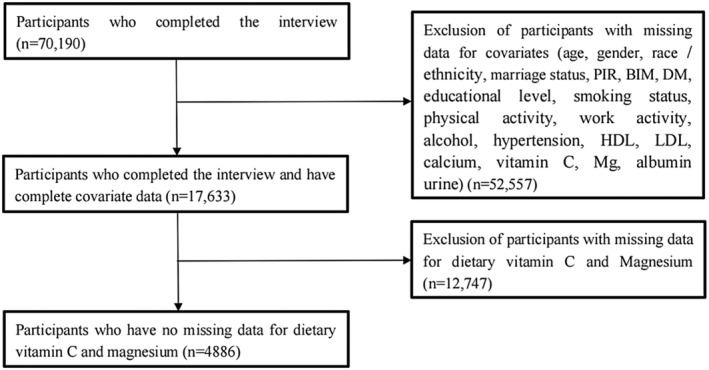
Flowchart of participants' enrollment in the study.

As depicted in Figure 1, after excluding participants with missing data, a total of 4886 participants with complete data were included for further analysis. Table 1 shows the descriptive characteristics of the participants' dietary Mg intake. Compared with normal dietary Mg intake group (≤254 mg/day), participants with low dietary Mg intake group (>254 mg/day) were more likely to be men, older, non Hispanic white, married, highly educated, high exercise intensity and work activity intensity, and to smoke and drink less. Individuals with high dietary Mg intake had higher PIR, normal levels of BMI, and they had higher intakes of vitamin C, dietary Ca, and less LDL and urinary albumin. There were no significant differences between the participants in the normal dietary Mg intake group and the low dietary Mg intake group with respect to whether they had hypertension and diabetes mellitus and high‐density lipoprotein content in the body (all *p* values > .05).

**TABLE 1:** Characteristics of participants.

Variables	Dietary magnesium intake			
	Total (*n* = 4886)	Mg ≤ 254 mg/day (*n* = 2431)	Mg > 254 mg/day (*n* = 2455)	*p*‐Value
Age, median (IQR)	39.0 (20.0, 60.0)	35.0 (18.0, 60.0)	42.0 (25.0, 59.0)	<.001
Gender, *n* (%)				
Female	2502 (51.2)	1472 (60.6)	1030 (42)	<.001
Male	2384 (48.8)	959 (39.4)	1425 (58)	
Race, *n* (%)				
Mexican American	953 (19.5)	432 (17.8)	521 (21.2)	<.001
Non‐Hispanic black	1166 (23.9)	728 (29.9)	438 (17.8)	
Non‐Hispanic white	1983 (40.6)	914 (37.6)	1069 (43.5)	
Other Hispanic	268 (5.5)	121 (5)	147 (6)	
Another race – including multi‐racial	516 (10.6)	236 (9.7)	280 (11.4)	
Married, *n* (%)				
Never married	1168 (23.9)	655 (26.9)	513 (20.9)	<.001
Married	3157 (64.6)	1409 (58)	1748 (71.2)	
Do not know	561 (11.5)	367 (15.1)	194 (7.9)	
Educational level, *n* (%)				
Less than high school	1800 (36.8)	1011 (41.6)	789 (32.1)	<.001
High school graduation	1027 (21.0)	561 (23.1)	466 (19)	
College or above	2059 (42.1)	859 (35.3)	1200 (48.9)	
BMI (kg/m^2^), *n* (%)				
<25	1832 (37.5)	936 (38.5)	896 (36.5)	<.001
25–29.9	1427 (29.2)	634 (26.1)	793 (32.3)	
>30	1627 (33.3)	861 (35.4)	766 (31.2)	
PIR, mean ± SD	2.5 ± 1.6	2.3 ± 1.5	2.7 ± 1.6	<.001
Work activity, *n* (%)				
Non‐work activity	981 (20.1)	464 (19.1)	517 (21.1)	<.001
Moderate work activity	447 (9.1)	214 (8.8)	233 (9.5)	
Vigorous work activity	483 (9.9)	206 (8.5)	277 (11.3)	
Do not know	2975 (60.9)	1547 (63.6)	1428 (58.2)	
Smoke status, *n* (%)				
Never smoked	2060 (42.2)	946 (38.9)	1114 (45.4)	<.001
Former smokers	965 (19.8)	421 (17.3)	544 (22.2)	
Current smokers	758 (15.5)	373 (15.3)	385 (15.7)	
Do not know	1103 (22.6)	691 (28.4)	412 (16.8)	
Had at least 12 alcoholic drinks/year, *n* (%)				
Yes	567 (11.6)	327 (13.5)	240 (9.8)	<.001
No	1237 (25.3)	498 (20.5)	739 (30.1)	
Do not know	3082 (63.1)	1606 (66.1)	1476 (60.1)	
Hypertension, *n* (%)				
No	3358 (68.7)	1672 (68.8)	1686 (68.7)	<.939
Yes	1528 (31.3)	759 (31.2)	769 (31.3)	
DM, *n* (%)				
No	3487 (71.4)	1756 (72.2)	1731 (70.5)	<.182
Yes	1399 (28.6)	675 (27.8)	724 (29.5)	
Low‐density lipoproteins (LDL), mean ± SD	106.8 ± 36.0	105.7 ± 36.5	107.9 ± 35.6	<.032
High‐density lipoproteins (HDL), mean ± SD	54.8 ± 15.4	54.5 ± 15.5	55.1 ± 15.4	.187
Vitamin C, nmol/L, Median (IQR)	0.9 (0.6, 1.2)	0.9 (0.6, 1.2)	1.0 (0.6, 1.2)	.001
Calcium, mg/day, mean ± SD	916.7 ± 582.3	641.1 ± 351.9	1189.5 ± 634.6	<.001
Mg, mg/day, mean ± SD	281.9 ± 146.4	283.1 ± 146.5	281.4 ± 146.3	.713
Albumin_urine, μg/ml, median (IQR)	9.2 (5.0, 18.6)	10.6 (5.7, 22.1)	7.9 (4.5, 15.5)	<.001

### 3.2 Correlation between dietary vitamin C content and eGFR

The correlation between dietary vitamin C concentrations and eGFR was thoroughly investigated, revealing a noteworthy positive relationship between dietary vitamin C content and eGFR in participants, as observed in the unadjusted models (*β*: 4.81; 95% CI: 2.96–6.67; *p* < .001) (Table 2). Moreover, after controlling for confounding variables, the positive association between participants' dietary vitamin C content and eGFR persisted (*β*: 3.1; 95% CI: 2.05 ~ 4.16; *p* < .001; Table 2).

**TABLE 2:** Association between dietary vitamin C and eGFR.

Models	eGFR (*n* = 4886)	
	*β* (95% CI)	*p*‐Value
Model1	4.81 (2.96 ~ 6.67)	<.001
Model2	3.85 (2.83 ~ 4.87)	<.001
Model3	3.57 (2.55 ~ 4.6)	<.001
Model4	3.65 (2.6 ~ 4.7)	<.001
Model5	3.25 (2.21 ~ 4.3)	<.001
Model6	3.1 (2.05 ~ 4.16)	<.001

*Note*: model1: not adjusted; model2: adjusted for age, gender, race; model3: model2 + married, PIR, BMI, education level, work activity; model4: model3 + alcohol use, smoking status; model5: model4 + albumin_urine, LDL, HDL, hypertension; model6: model4 + dietary calcium intake, dietary magnesium intake.

### 3.3 Magnesium intake affects the link between dietary vitamin C and eGFR

The analysis was adjusted for various factors, including age, sex, race, marital status, PIR, BMI, education level, work activity, alcohol consumption, smoking status, urinary albumin, LDL, HDL, hypertension, and dietary calcium intake. There was an interaction between dietary magnesium and dietary vitamin C on the relationship between eGFR (Table 3). Participants' dietary vitamin C intake was categorized into two groups based on its content: a low‐level group and a high‐level group. In the low dietary Mg intake group (≤254 mg/day), the mean eGFR of participants in the high dietary vitamin C intake group was 2.96 (95% CI:1.63 ~ 4.29，*p*<.001), and showed that dietary Mg and vitamin C intake had a correlation with eGFR (interaction likelihood ratio test ¦ The value is <.05). However, in the normal dietary Mg intake group (>254 mg/day), the association was not significant (*β* = 1.05, 95% CI: 0.15 ~ 2.25, *p* = .085).

**TABLE 3:** Interactive effects of dietary vitamin C and dietary magnesium intake on eGFR.

Variable	Magnesium ≤ 254 (mg/day) (*n* = 2431)		Magnesium > 254(mg/day) (*n* = 2455)		*p* For interaction
	*β* (95% CI)	*p*‐Value	*β* (95% CI)	*p*‐Value	
Subgroups					
Low‐level	0 (Reference)		0 (Reference)		.020
High‐level	2.96 (1.63 ~ 4.29)	<.001	1.05 (−0.15 ~ 2.25)	0.085	

*Note*: Adjusted for age, gender, race; marital status, PIR, BMI, education level, work activity; alcohol use, smoking status; albumin_urine, LDL, HDL, hypertension; and dietary calcium intake.

## 4 DISCUSSION

Our analysis of data from a representative adult population in the United States revealed a significant positive correlation between participants' dietary vitamin C intake and eGFR. Moreover, we observed an interaction between dietary magnesium intake and dietary vitamin C content, which had a greater impact on eGFR than the combined individual effects. Further studies showed that this interaction was more significant at low dietary Mg intakes compared to normal dietary Mg intakes.

To the best of our knowledge, this is the first large‐scale study to investigate the association between dietary magnesium intake and vitamin C intake with eGFR. Consistent with our findings, a previous meta‐analysis comprising four RCTs reported that antioxidant therapy significantly enhanced kidney function (Jun et al., 2012). The primary mechanism underlying the kidney's protective effect of vitamin C is attributed to its ability to scavenge oxygen‐free radicals. Oxidative stress is a known factor in renal injury (Levey & James, 2017). In addition, vitamin C is implicated in pathophysiological processes related to ischemia–reperfusion, immunomodulation, and inflammation. These conditions are closely associated with the kidney function (Honore et al., 2020). In light of these findings, it is plausible that obstructing the oxidative stress pathway with other antioxidant vitamins may also mitigate renal injury (Mune et al., 2002; Schnackenberg, 2002; Van den Branden et al., 2002). Furthermore, a mouse experiment revealed that the addition of vitamin C and vitamin E could alleviate renal injury associated with malignant hypertension by reducing the release of renal superoxide and mitigating tissue inflammation (Tian et al., 2005). In summary, these findings collectively support the notion that vitamin C intake could enhance eGFR in both healthy individuals and those with renal dysfunction.

Magnesium could mitigate vascular smooth muscle cells apoptosis and higher phosphate‐induced vascular calcification by binding phosphate or reducing serum phosphorus levels (Sakaguchi et al., 2015). A retrospective cohort study comprising 311 non‐dialysis CKD patients demonstrated that those in the high phosphate and low magnesium groups had a higher risk of ESKD than those in the high phosphate and high magnesium groups (Sakaguchi et al., 2015). Moreover, hypomagnesemia contributes to complications, such as DM and graft loss, after kidney transplant (Sakaguchi et al., 2015).

Surprisingly, this study provided a summary of the association between dietary vitamin C and dietary magnesium with eGFR. The results indicated a significant positive correlation between dietary vitamin C intake and eGFR, which was notably stronger in individuals with low dietary magnesium intake. Nevertheless, the underlying mechanism of the interaction between magnesium and vitamin C on eGFR warrants further investigation. Interestingly, a rat model experiment showed that magnesium and vitamin C could inhibit TNF‐*α* cell expression by cooperative reducing oxidative damage in vivo (Zheng et al., 2020). Coincidentally, the rats' experiment had found that the combination of vitamin C and magnesium ions can significantly promote the M2 polarization of macrophages in tissues, further inhibiting the expression of pain‐related neuropeptides (Yao et al., 2021). A double‐blind RCT demonstrated that the combination of magnesium and vitamin C could synergistically alleviate postoperative pain and reduce pain scores (Moon et al., 2020). Furthermore, magnesium ions supplementation could increase the cellular uptake of vitamin C, further enhancing the anticancer effects of vitamin C in cancer cells (Cho et al., 2020). These reported mechanisms might be involved in the synthetic kidney protective role of vitamin C and magnesium.

This study also has some limitations. Firstly, the presence of unmeasured or residual confounding factors cannot be entirely ruled out, despite the various adjustments made in the study. Secondly, the participants' magnesium intake was obtained through a questionnaire, which may have been subject to recall bias. Additionally, the use of self‐reported 24‐hour dietary recalls to determine dietary intake may have led to an underestimation of nutritional intake, and the overall energy intake was not evaluated. Some potential effects on nutritional intake, including specific drugs (e.g., dietary supplements) and recommendations from clinicians from participants, were also not considered in this study. Thirdly, while a large sample was included in the study, the population under investigation was limited to US residents. As such, caution should be exercised when extrapolating these findings to other populations or samples. However, the strengths of this study include its large population size, comprehensive scope, and inclusion of important covariates related to the same.

## 5 CONCLUSION

Based on our research findings, there is a positive correlation between dietary vitamin C intake and eGFR, which is particularly pronounced in individuals with low dietary magnesium intake. The interaction between dietary magnesium and the relationship between vitamin C and glomerular filtration rate could hold significant implications for the clinical use of drugs aimed at preventing diseases associated with abnormal glomerular filtration rates.

## AUTHOR CONTRIBUTIONS

**Zheng‐yang Lin:** Conceptualization (lead); data curation (equal); formal analysis (equal); project administration (equal); writing – original draft (equal); writing – review and editing (equal). **Yong‐yi Liang:** Conceptualization (equal); data curation (equal); formal analysis (equal); resources (equal). **Ru Wang:** Conceptualization (equal); writing – original draft (equal); writing – review and editing (equal). **Biao Hu:** Data curation (equal); software (equal). **Wen‐ju He:** Supervision (equal); validation (equal). **Jun‐kui Li:** Software (equal). **Zi‐ang Ding:** Writing – original draft (equal). **Zhuo‐yuan Lin:** Resources (equal). **Shi Zhang:** Resources (equal).

## CONFLICT OF INTEREST STATEMENT

All authors declare no competing interests.

## Data Availability

The data that support the findings of this study are available in NHANES at [https://wwwn.cdc.gov/nchs/nhanes/continuousnhanes/].
